# Collagen Membrane: A Viable Option for Oral Soft Tissue Defects—A Case Series

**DOI:** 10.1002/ccr3.70349

**Published:** 2025-04-02

**Authors:** Niroj Khanal, Bibek Kattel, Mehul Rajesh Jaisani, Siddhartha Rai

**Affiliations:** ^1^ Department of Oral and Maxillofacial Surgery BP Koirala Institute of Health Sciences Dharan Nepal; ^2^ College of Dental Surgery BP Koirala Institute of Health Sciences Dharan Nepal

**Keywords:** collagen sheath, dysplasia, leukoplakia, oral mucosa, oral soft tissue defects

## Abstract

Timely intervention during the predysplastic or dysplastic stages of red and white lesions is crucial for preventing their progression to oral carcinoma. Following surgical removal, a soft tissue deficit area is created. Surgical excision of these lesions often leads to a mucosal defect. These residual defects can be concealed with skin grafts, mucosal grafts, or xenografts. In this paper, we describe collagen sheets as an excellent reconstruction option, particularly after the loss of soft tissue due to resection of red and white lesions with histologically verified dysplastic features.

## Graphical abstract

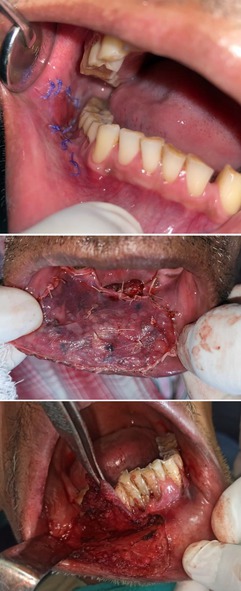

**Summary**

- Routine oral examinations and timely biopsy of suspicious lesions are crucial for early diagnosis and treatment, potentially averting the progression to cancer. Both patients and healthcare providers should be vigilant about oral health, especially in individuals with risk factors, such as prolonged tobacco use.

## 1 Introduction

Leukoplakia, along with its variants erythroplakia and submucous fibrosis (which is particularly prevalent in the Indian subcontinent), is a condition strongly linked to the development of oral epithelial dysplasia and eventually, oral squamous cell carcinoma. Because of its potential for malignant transformation, this condition represents a critical area of concern within the oral cavity. Close observation and timely intervention in patients with these conditions are needed to mitigate the risk of progression to more severe forms of oral cancer [1].

The frequency of malignant transformation of dysplastic lesions is 15.6%–39.2% in several studies. In a significant retrospective study examining 3300 biopsies of oral white lesions, Waldron and Shafer found that 19.9% of leukoplakia exhibited varying degrees of epithelial dysplasia [2, 3].

Histopathologically, leukoplakia shows hyperkeratosis accompanied occasionally by acanthosis, with epithelial atrophy in some cases and various inflammatory cells in the connective tissue. The dysplasia upon biopsy is seen in 5%–25% of the cases when all oral sites are explored, often beginning in the basal epithelial layers [3].

Various techniques, including oral mucosa free grafts, oral connective tissue grafts, and split and full‐thickness skin grafts, have traditionally been employed for treating precancerous lesions. Tissue engineered grafts are the most recent advancement in this field. Attempts have been made to use free microvascular grafts or pedicled local and distant flaps for larger defects with varying success rates [4].

The surgical excision of a precancerous lesion can result in substantial loss of soft tissue that may not be suitable for primary closure [1].

Other alternative options for reconstruction that are being used widely in burn cases and nowadays in the surgical defect are xenogenous collagen sheets, as the raw areas of excised premalignant lesions are too large to be closed primarily [5, 6, 7].

Skin constitutes over 80% of collagen, which is the most predominant insoluble fibrous protein in connective tissues. In the early 1970s, John F. Burke and Ioannas Yannas pioneered a biocompatible collagen matrix to enhance wound healing [8]. This innovation finds common application in management of burn [9], diabetic foot ulcer [7], toxic epidermal necrolysis [10], chronic wounds, etc. [11]

This versatile material is also used in the field of oral and maxillofacial surgery and general dentistry. It serves various purposes, such as an interpositional graft material during palatoplasty [12] and guided bone regeneration during maxillary sinus lift inducing bone formation. Additionally, it is used for treating localized gingival recession, as a reconstructive material for fractures of the orbital floor, for bone augmentation of the posterior atrophic mandibular ridge to facilitate dental implant placement [13], as a scaffold in tissue engineering to generate dental pulp regeneration [14], and for the coverage of minor intraoral soft tissue defects and much more [6].

Through case series, we describe the use of collagen membrane—a viable alternative for reconstruction of these limited‐sized mucosal defects.

## 2 Case Presentation

### 2.1 Patient 1

#### 2.1.1 History and Clinical Examination

**FIGURE 1:**
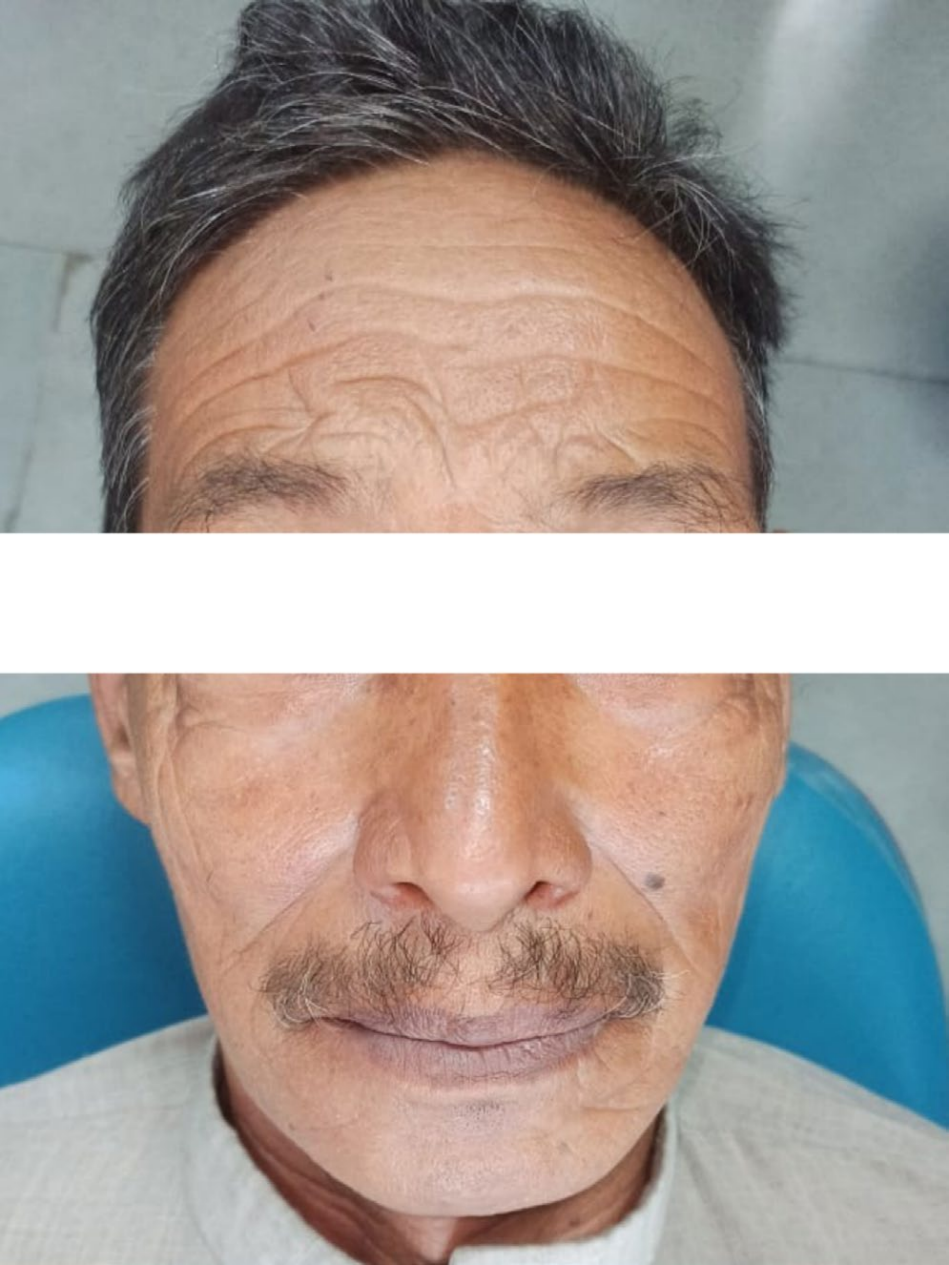
Extraoral photograph of patient.

A 69‐year‐old male patient (Figure 1) reported to the department of oral and maxillofacial surgery with the chief complaint of a whitish patch present on the right labial mucosa and vestibule for 6 months, with a noncontributory medical history. The patient had the habit of consuming chewable tobacco five to six times per day for the last 40 years and had left the habit for the last 5 months.

**FIGURE 2:**
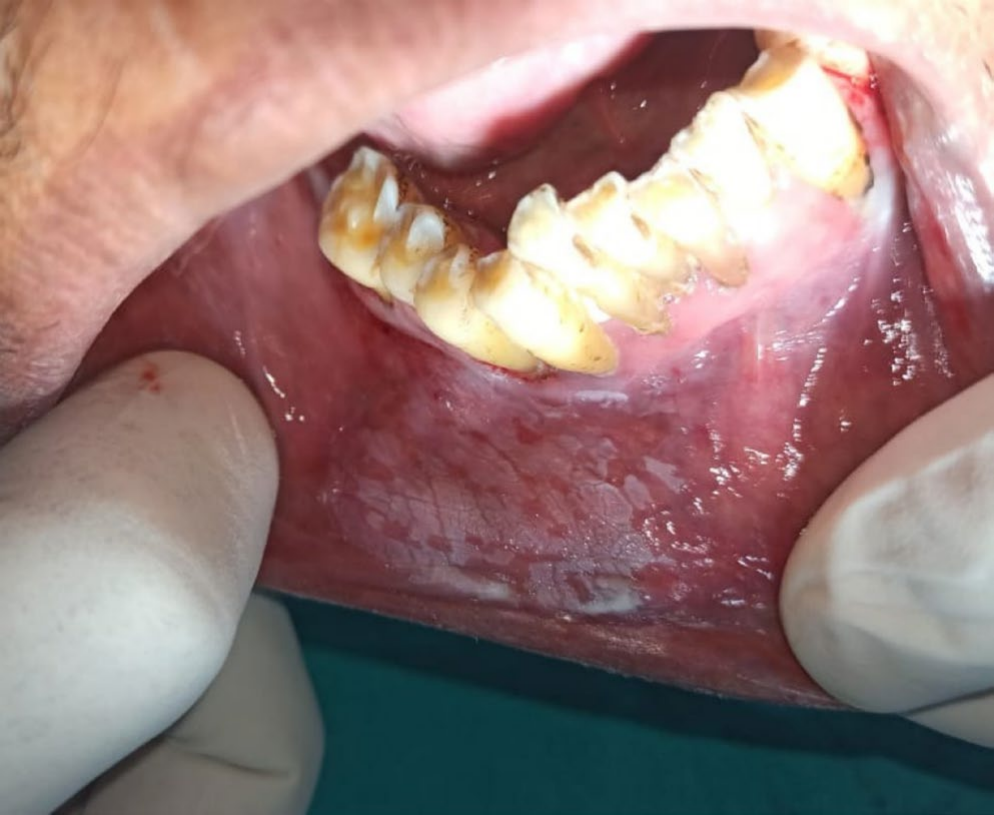
Intraoral photograph showing whitish patch on lower right labial and buccal mucosa and vestibule in relation to tooth numbers 42, 43, 44, and 45.

Intraoral examination revealed discrete, nonscrapable whitish keratotic patches with cracked mud texture without striae measuring approximately 3 cm × 3 cm in size over the lower right labial and buccal mucosa extending to the vestibule in relation to tooth numbers 42, 43, 44, and 45 (Figure 2).

Based on the history and clinical examination, a provisional clinical diagnosis of oral leukoplakia was made. However,

#### 2.1.2 Investigations

Histopathological examination of the incisional biopsy specimen was suggestive of severe dysplasia.

#### 2.1.3 Treatment

**FIGURE 3:**
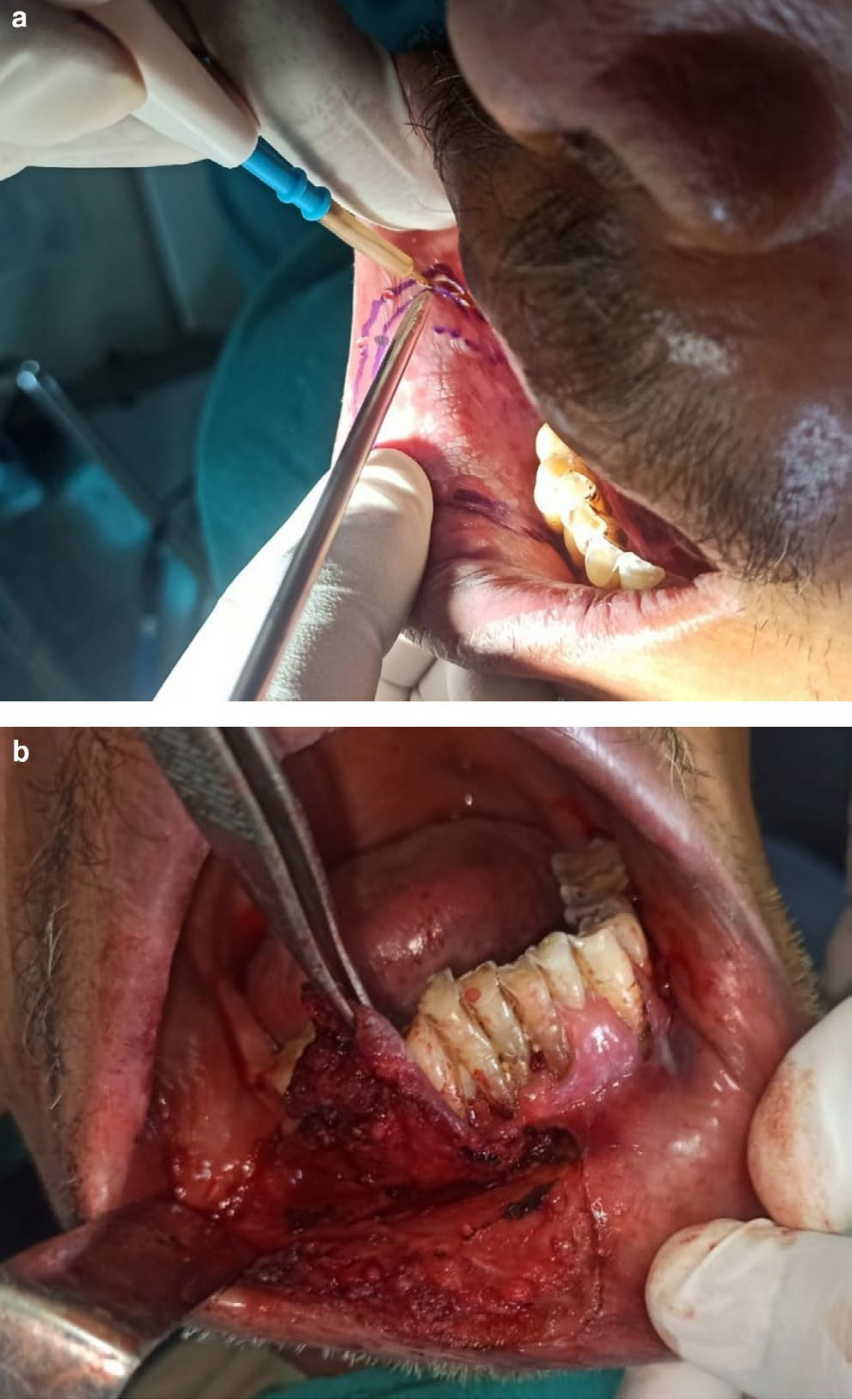
(a) Surgical excision of lesion using electrocautery. (b) Intraoperative photograph.

**FIGURE 4:**
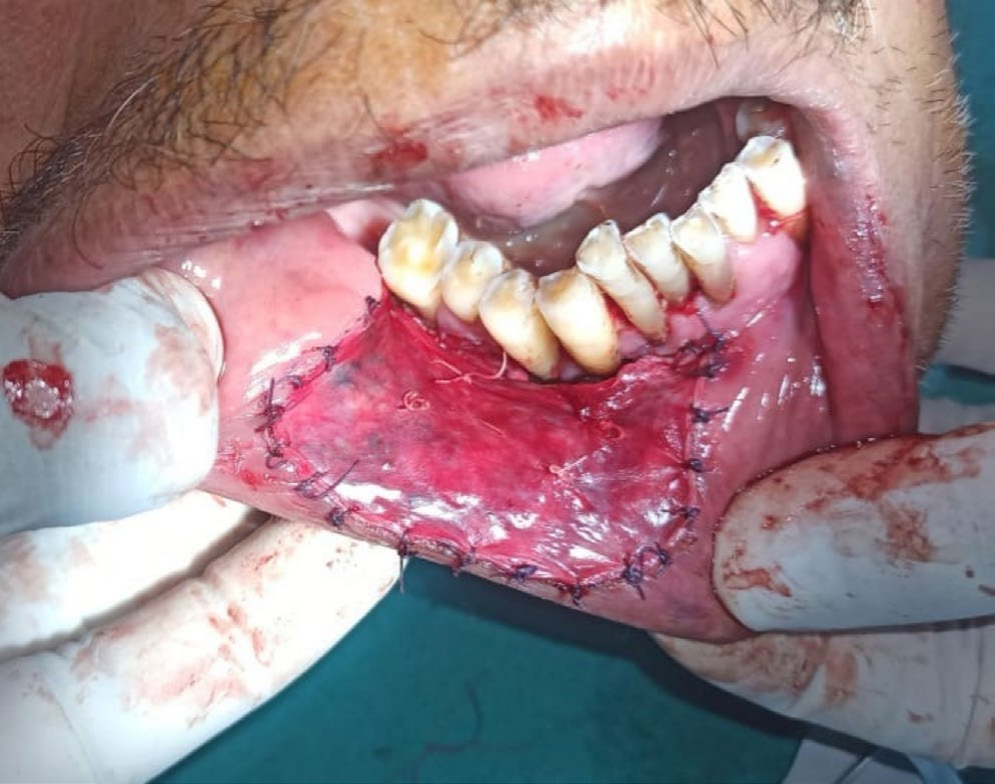
Soft tissue defect repair using collagen sheath.

**FIGURE 5:**
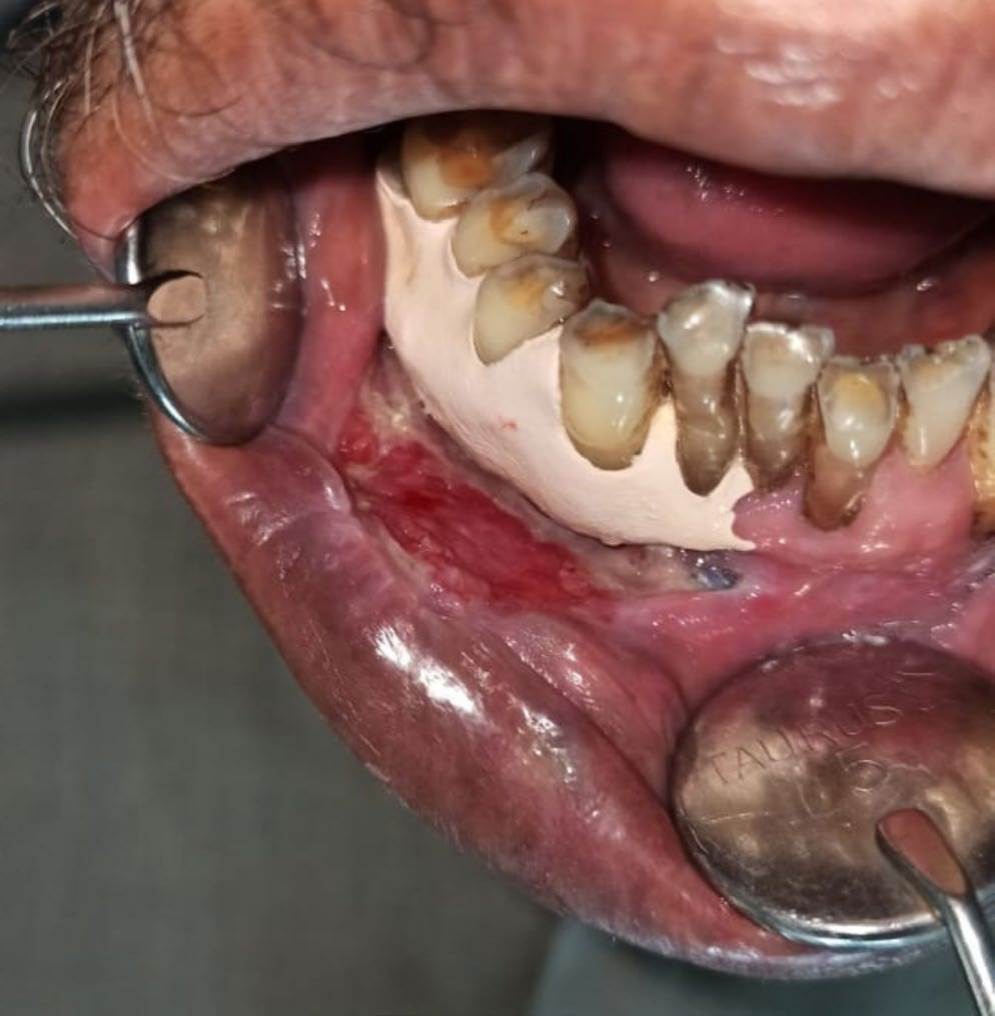
Intraoral postoperative photograph: after 7th day of surgery, showing satisfactory wound healing and zinc oxide eugenol periodontal dressing in situ.

**FIGURE 6:**
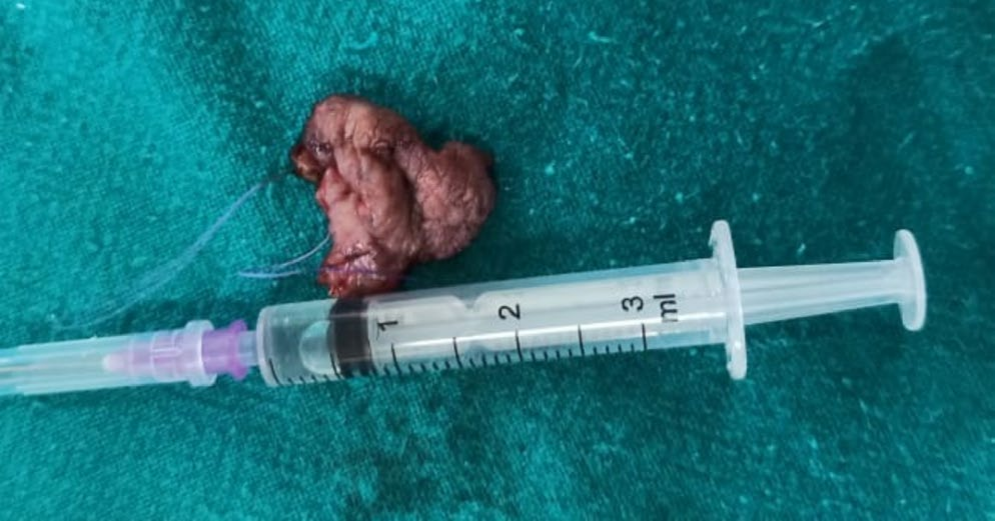
Excised dysplastic tissue for repeated HPE.

The lesion was surgically excised in toto using standard surgical protocol by taking a 5 mm healthy margin. Local anesthesia via inferior alveolar nerve block, buccal nerve block, and mental nerve block; electrocautery; and scalpel were used for excision. Dissection was carried out in the supramuscular plane with the preservation of the mental nerve (Figure 3). The soft tissue defect then created was covered using a collagen sheath/membrane and secured with 4–0 vicryl (Figure 4). The raw area over the alveolar bone was covered with zinc oxide eugenol periodontal dressing (Figure 5). The excised tissue was oriented (Figure 6) and sent for histopathological reevaluation, which confirmed the previously made diagnosis.

#### 2.1.4 Outcome and Follow‐Up

Patient was advised to have a liquid diet for 2 weeks. The following oral antibiotics were given: amoxicillin 500 mg three times a day for 5 days, metronidazole 400 mg three times a day for 5 days, an analgesic etoricoxib 90 mg once daily for 3 days, and proton pump inhibitors, pantoprazole 40 mg, once daily were given for 5 days. Postoperatively, the patient was asked to rinse the mouth with 0.2% chlorhexidine after 24 h. Periodontal dressing was removed on postoperative day 10. Later, the subsequent follow‐up was maintained at 1 week, 2 weeks, 1 month, 3 months, and 6 months, during which there was satisfactory wound healing with no obliteration of vestibular depth.

**FIGURE 7:**
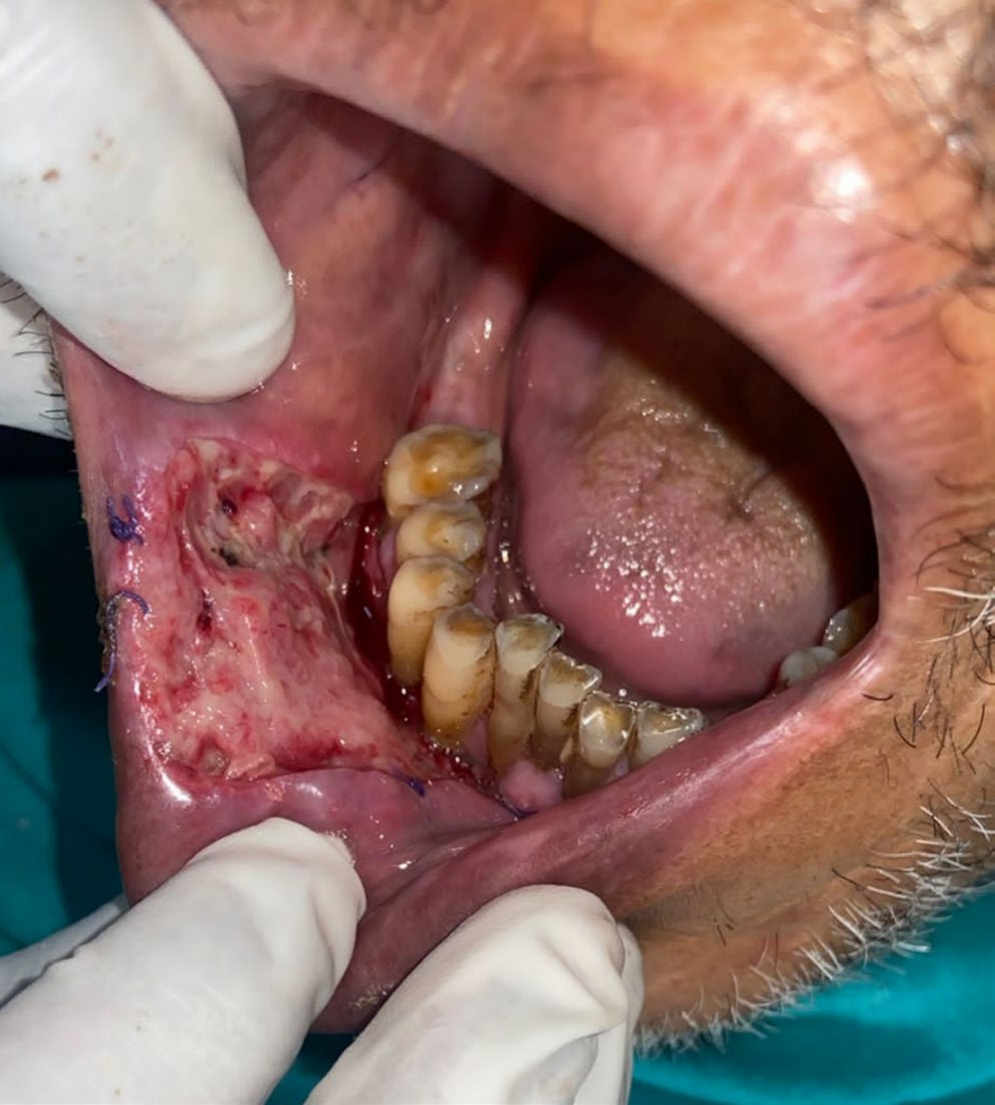
Intraoral postoperative photograph: after 14th day of surgery, showing satisfactory wound healing and zinc oxide eugenol periodontal dressing was removed.

**FIGURE 8:**
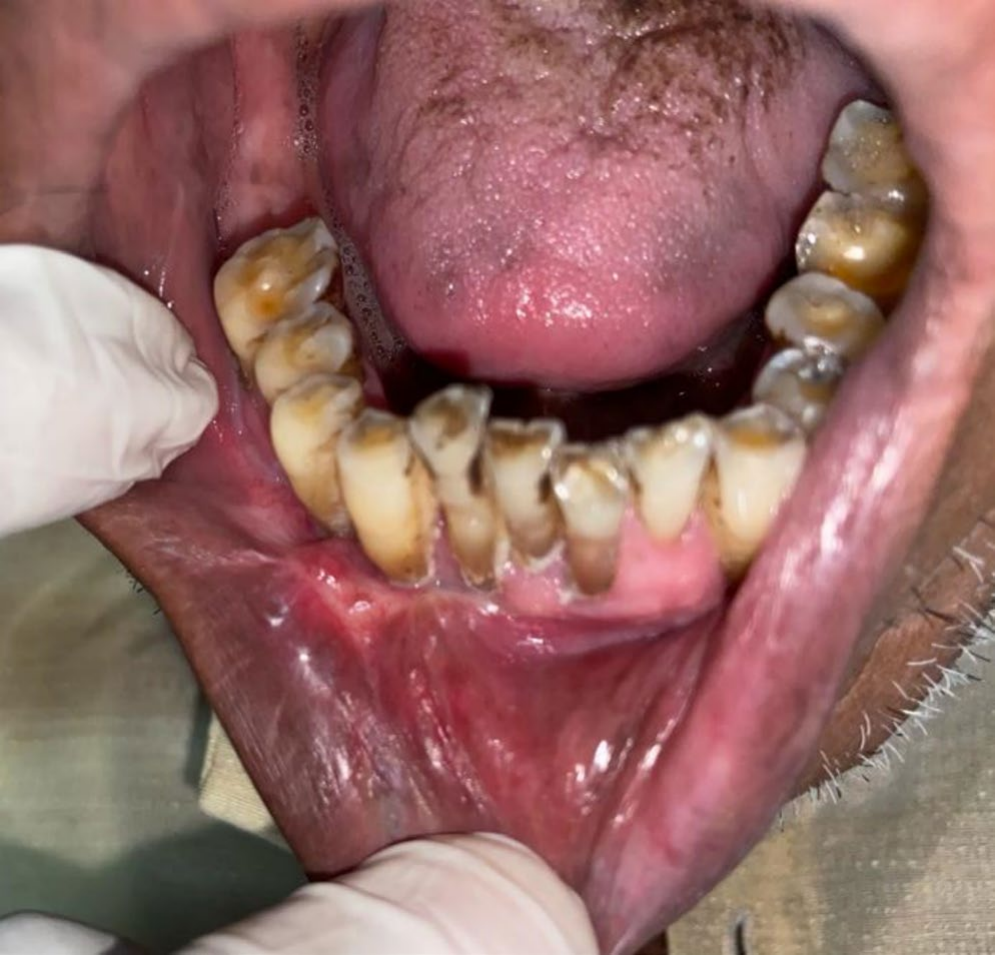
Intraoral postoperative photograph: 1 month after surgery, showing satisfactory wound healing with maintained vestibular depth.

**FIGURE 9:**
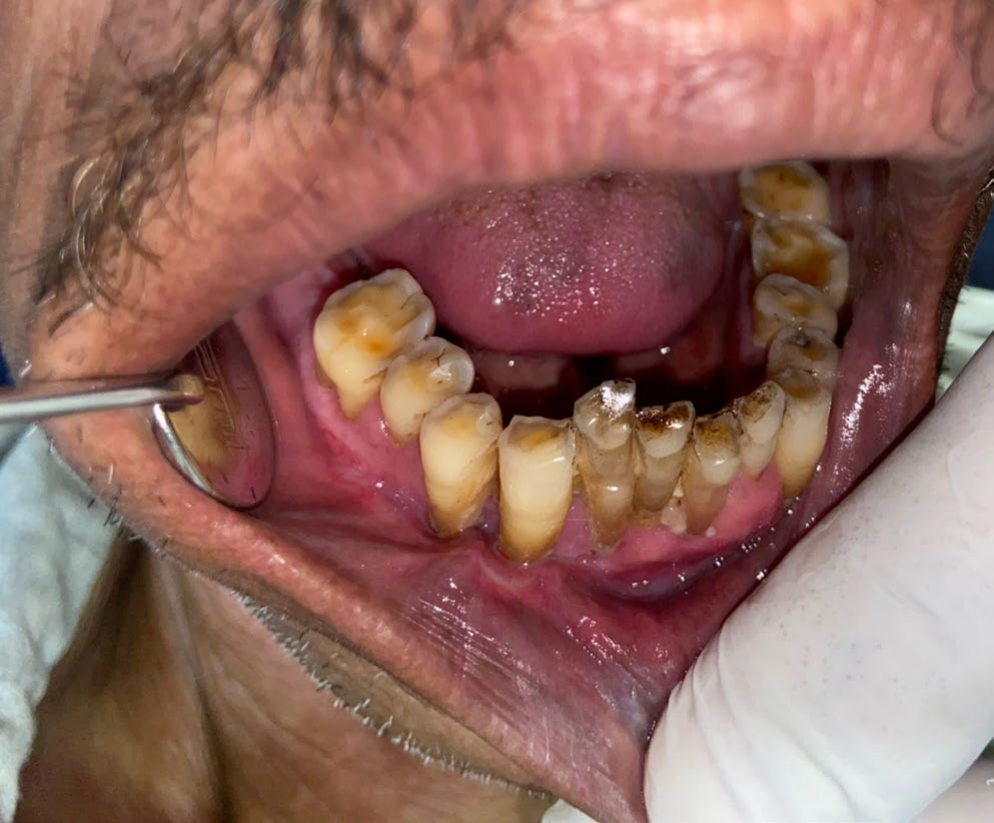
Intraoral postoperative photograph: 3 months after surgery, showing normal mucosa.

There was satisfactory wound healing with no obliteration of vestibular depth and no signs of local recurrence (Figures 7, 8, 9).

Periodontal dressing was extended into the vestibule and removed only after postoperative day 10 to avoid vestibular obliteration.

### 2.2 Patient 2

#### 2.2.1 History and Clinical Examination

**FIGURE 10:**
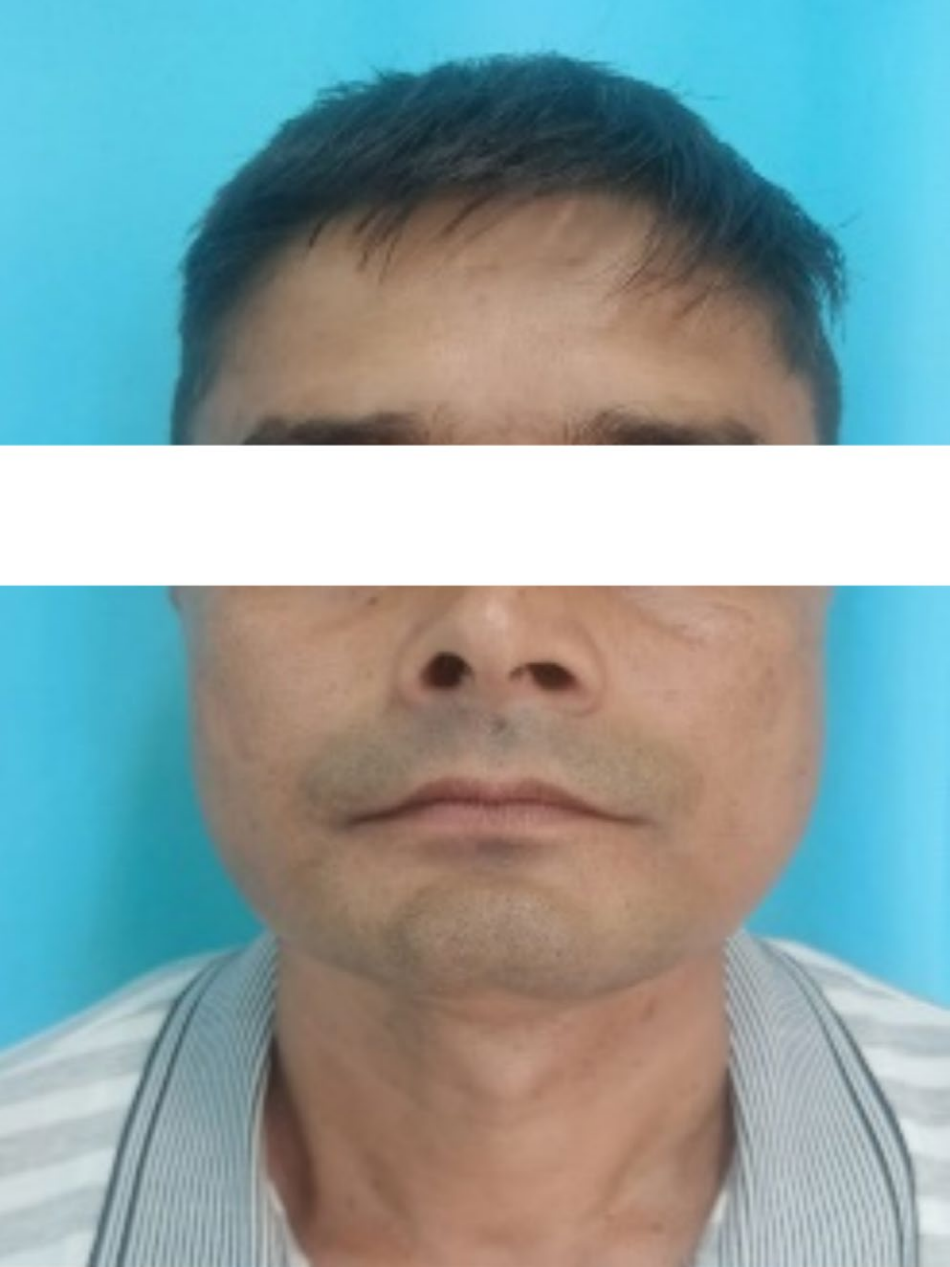
Extraoral photograph of the patient.

A 49‐year‐old male patient (Figure 10) reported to the department of oral and maxillofacial surgery with the chief complaint of a white patch on the lower right posterior region of the mouth since 4 months. It was insidious in onset, gradually increasing in size with no pain during manipulation and an occasional burning sensation on the consumption of spicy food. His past medical history, including his family history, was unremarkable.

Patient was a tobacco chewer for the last 20 years, five to seven times per day.

**FIGURE 11:**
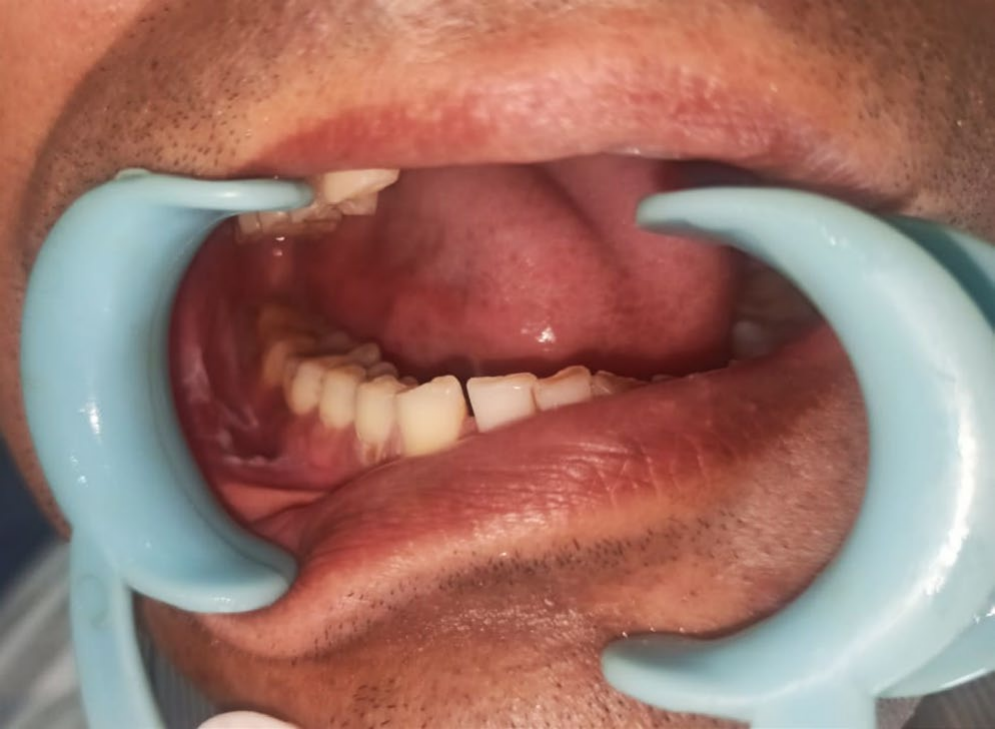
Clinical photograph of intraoral lesion.

There was no cervical lymphadenopathy. Intraoral examination revealed discrete, nonscrapable, nontender white keratotic patches with a cracked mud texture without striae, measuring 3 cm × 3 cm in size on the mandibular gingivobuccal sulcus with respect to tooth numbers 44–48 (Figure 11).

Based on the history and clinical examination, a provisional clinical diagnosis of oral leukoplakia was made.

#### 2.2.2 Investigations

Incisional biopsy from the lesion was suggestive of moderate dysplasia.

#### 2.2.3 Treatment

Following histological diagnosis, the lesion was surgically excised with a 0.5‐cm healthy margin. The soft tissue defect was covered using the collagen sheath/membrane and secured with sutures. The raw area over the alveolar side was covered with zinc oxide eugenol periodontal dressing. A Ryle's tube was placed for feeding and maintained for 2 weeks. The excised tissue was oriented using sutures and then sent for histopathological reevaluation, which confirmed the previously made diagnosis.

#### 2.2.4 Outcome and Follow‐Up

**FIGURE 12:**
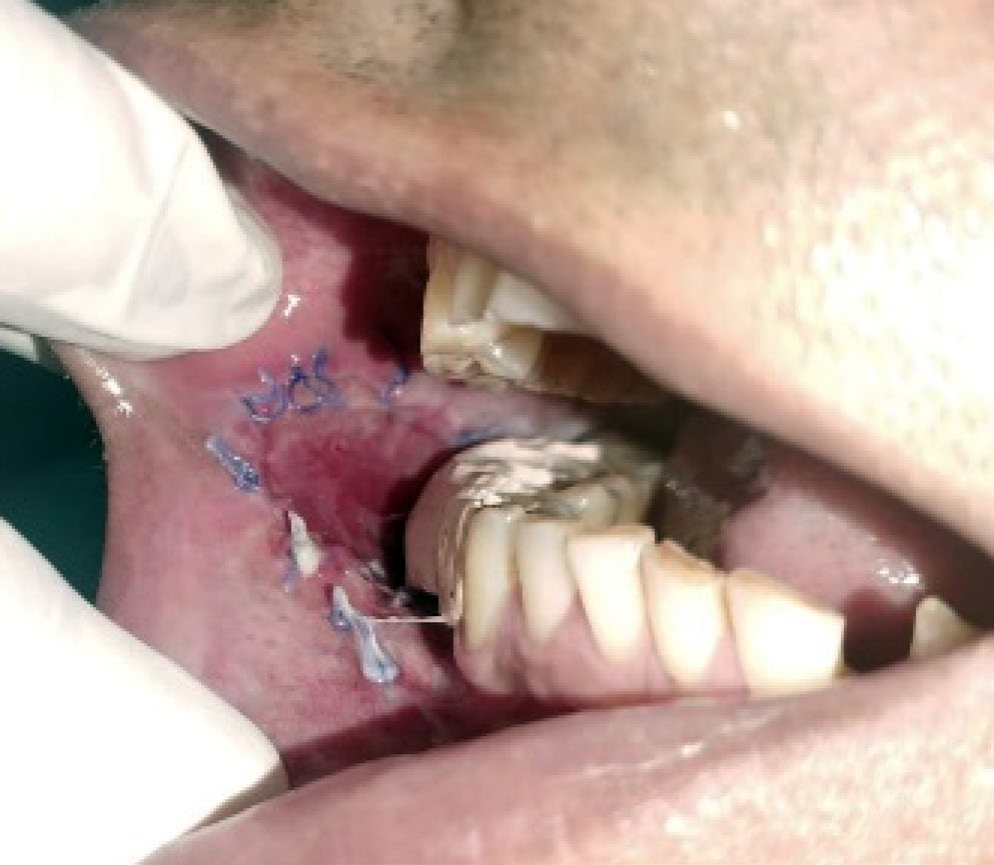
Seventh day postoperative photograph.

**FIGURE 13:**
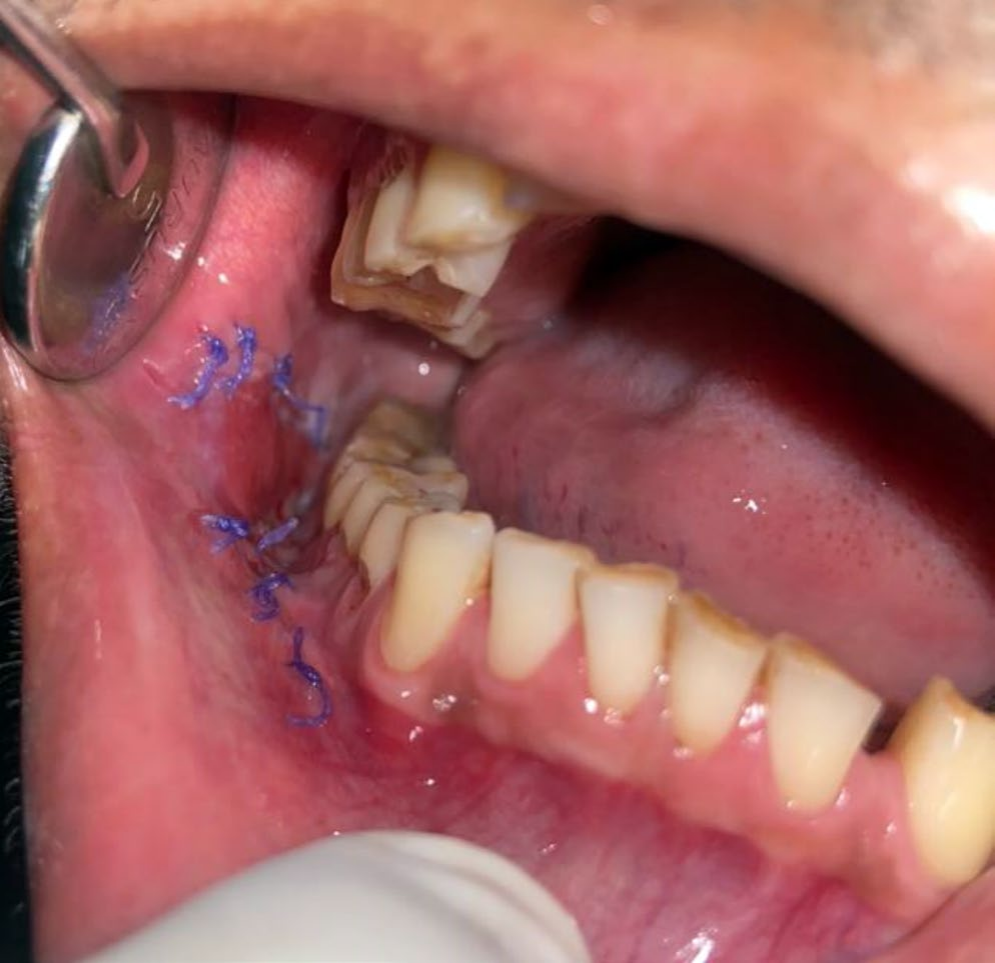
Fourteenth day postoperative photograph.

**FIGURE 14:**
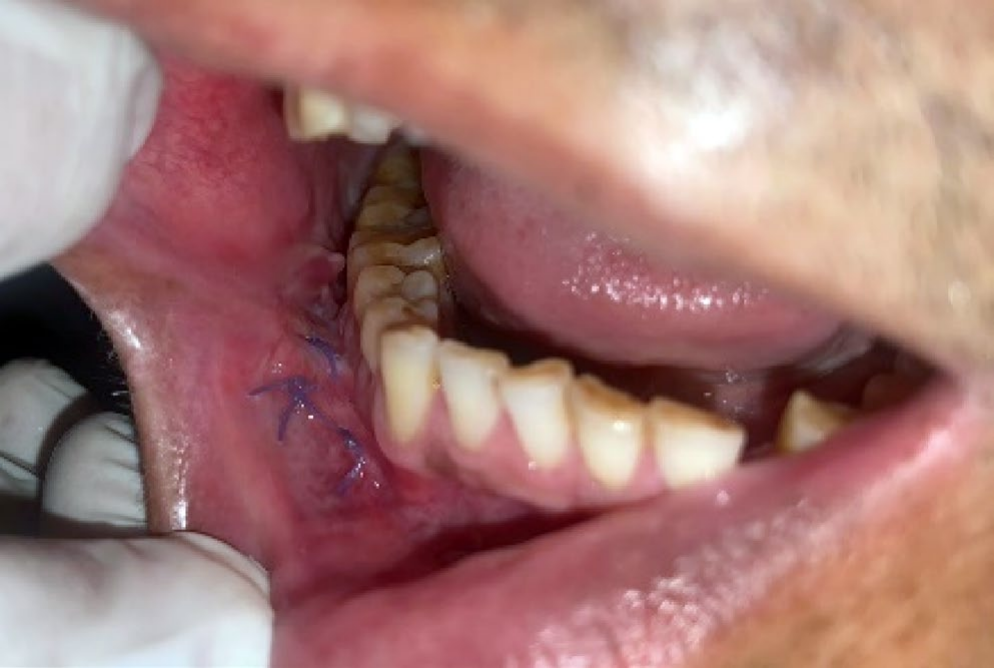
At 1‐month follow‐up.

The following oral antibiotics were given (per NG): amoxicillin 500 mg three times a day for 5 days, metronidazole 400 mg three times a day for 5 days, analgesic etoricoxib 90 mg once daily for 3 days, and proton pump inhibitors, pantoprazole 40 mg, once daily for 5 days. Patient was asked to rinse mouth with 0.2% chlorhexidine after 24 h. Zinc oxide‐eugenol periodontal dressing was removed on postoperative day 10. Patient was followed up at 1 week, 2 weeks, 1 month, 3 months, and 6 months. We observed a satisfactory wound healing with no obliteration of vestibular depth (Figures 12, 13, 14).

### 2.3 Patient 3

#### 2.3.1 History and Clinical Examination

**FIGURE 15:**
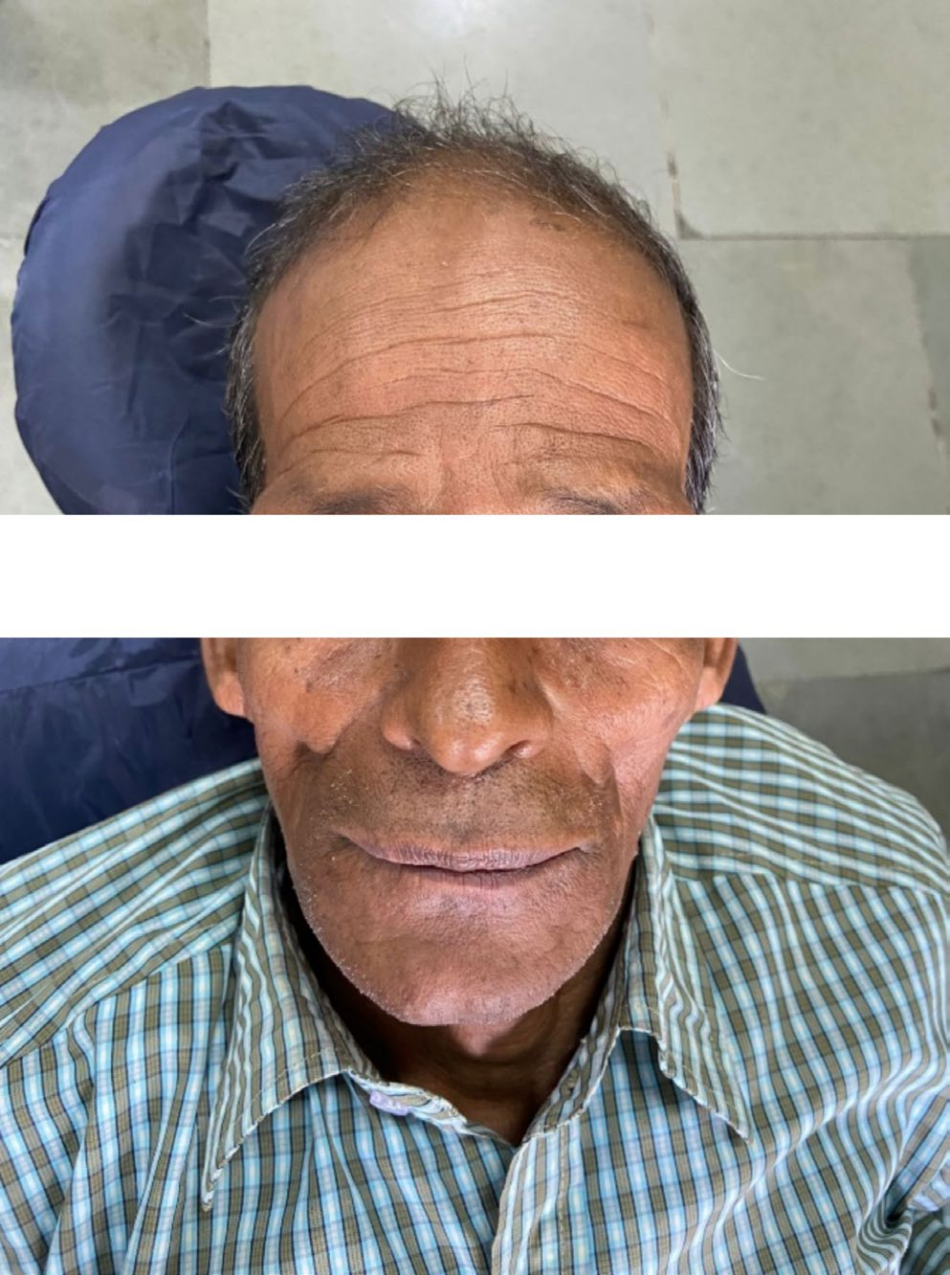
Extraoral photograph of patient.

A 63‐year‐old man (Figure 15) reported to the department of oral and maxillofacial surgery with the chief complaint of a white patch over the right labial mucosa and alveolar ridge for 8 months. His past medical history, including his family history, was unremarkable.

**FIGURE 16:**
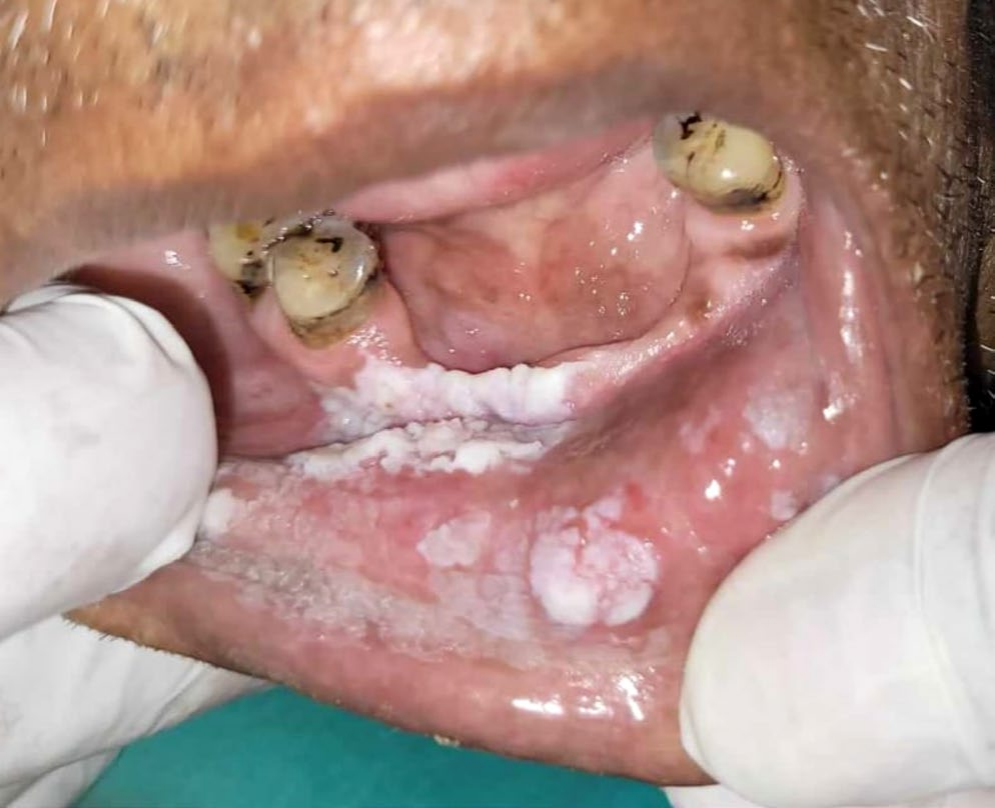
Intraoral photograph showing whitish patch on mandibular anterior alveolus in relation to tooth numbers 41–44, extending to vestibule, including labial frenum.

Patient gave a history of tobacco chewing for 20 years, five to seven times per day, but has quit the habit completely for the last few months. Intraoral examination revealed discrete whitish keratotic patches with cracked mud texture without striae measuring 4 cm × 3 cm in size present on the mandibular anterior alveolus in relation to 41–44, extending to vestibule, including the labial frenum, and was nonscrapable, non‐tender on palpation (Figure 16). It was insidious in onset, gradually increasing in size with no pain during manipulation and an occasional burning sensation on consumption of spicy food. Based on the history and clinical examination, a provisional clinical diagnosis of oral leukoplakia was made.

#### 2.3.2 Investigations

Incisional biopsy from the right mandibular alveolus region was suggestive of moderate dysplasia.

#### 2.3.3 Treatment

**FIGURE 17:**
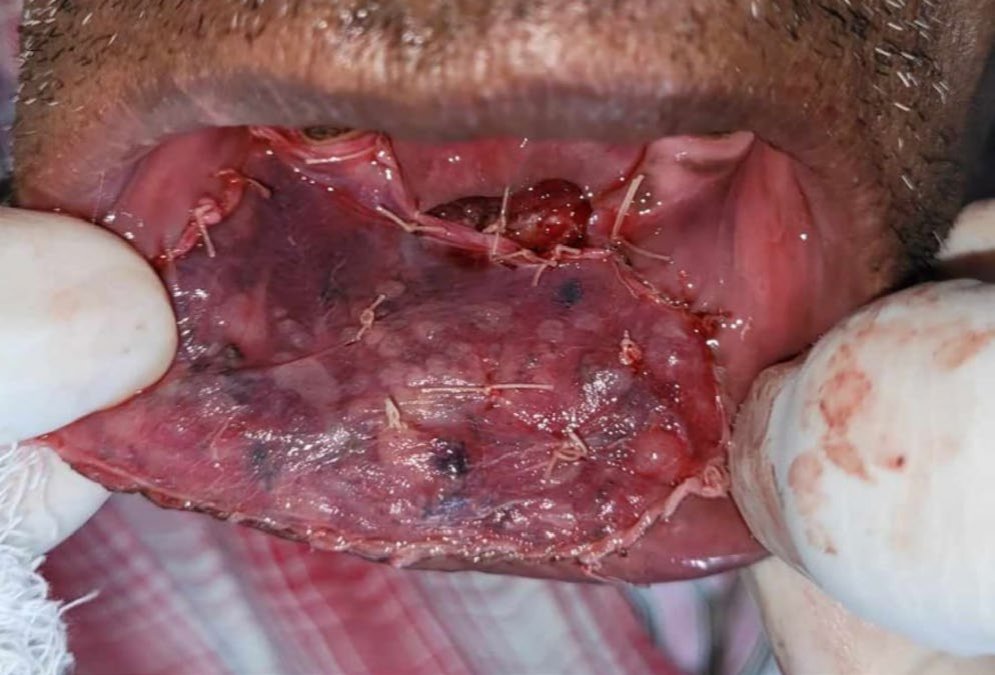
Soft tissue defect repair using collagen sheath.

Following histological diagnosis, the lesion was surgically excised into using standard surgical protocol by taking a 0.5 ‐cm healthy margin. Dissection was carried out in the supramuscular plane with the preservation of the mental nerve. The soft tissue defect was covered using the collagen sheath (Figure 17) and secured with sutures. The raw alveolar area was covered with zinc oxide eugenol periodontal dressing.

#### 2.3.4 Outcome and Follow‐Up

**FIGURE 18:**
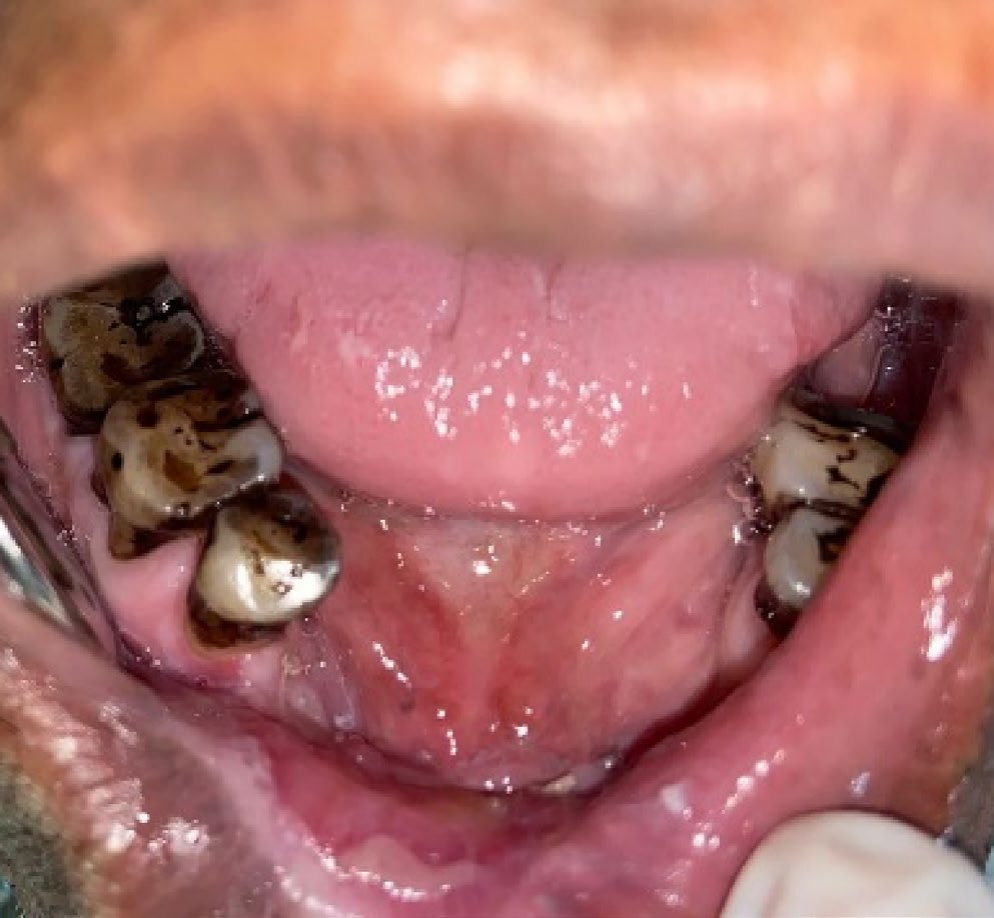
Intraoral 1 month postoperative photograph, showing healing.

**FIGURE 19:**
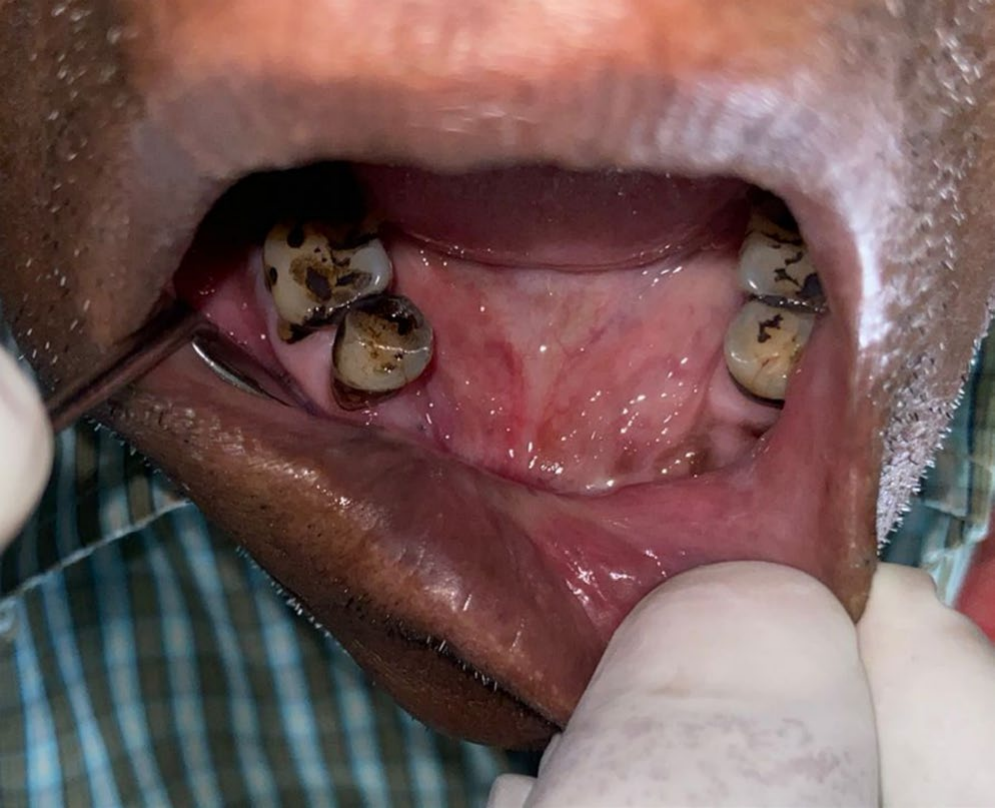
Intraoral 3 months postoperative photograph, showing normal mucosa.

Similar postoperative regime was followed. Regular follow‐ups were maintained. We observed satisfactory mucosalization with no vestibular obliteration (Figures 18 and 19).

## 3 Postoperative Care

Postoperative monitoring after oral precancerous lesion excision with collagen sheet includes assessing wound healing, preventing infection, and managing pain and inflammation. Regular follow‐ups (1, 3, and 6 months) ensure proper collagen integration, mucosal regeneration, and early detection of recurrence, especially in high‐risk patients. Patients should maintain strict oral hygiene, avoid irritants, and follow lifestyle modifications, including tobacco and alcohol cessation, to optimize healing and prevent recurrence. The patients physically visited the outpatient department for follow‐ups for up to 3 months postdischarge. They were thoroughly counseled about potential danger signs, including infection and recurrence. Because they resided in remote and distant areas of eastern Nepal, further follow‐ups were conducted via telecommunication, allowing us to maintain surveillance over their condition for several months.

## 4 Discussion

Regardless of age and gender, surgical excision of oral lesions was determined on the basis of specific criteria, including histopathologically confirmed moderate‐to‐severe dysplasia, unifocal lesions larger than 2 cm in diameter regardless of location, and a history of tobacco use or alcohol consumption. Additionally, lesions that failed to heal with conservative management or those that were long‐standing and persistent were considered for surgical intervention [15].

According to Ashley's principles of plastic surgery, prompt coverage of a raw wound is essential to prevent infection, tissue contracture, and scarring. In cases of extensive or irreversible damage to soft tissue, immediate coverage using skin substitutes becomes necessary to facilitate repair and regeneration [16].

Healing is a complex dynamic process that culminates in the restoration of both anatomical continuity and function. This intricate process typically follows a systematic and biologically orchestrated series of events. Healed wounds constitute a spectrum of repair, and they must be defined and specified.

Ideally healed wound embodies a restoration to the normal state of anatomic structure, function, and appearance. It signifies the successful culmination of the healing process [14].

Using these grafts requires an additional surgical procedure, posing technical challenges. Skin grafts, in particular, do not fully match the color and texture of the oral cavity and may lead to the growth of adnexal structures, such as hair and sweat glands. Additionally, in elderly patients, the skin tends to be atrophic and inelastic, making it less suitable for grafting. Furthermore, the dynamic and mobile nature of the oral environment poses obstacles to successful graft acceptance [6, 7, 17].

For over a century, the understanding that grafted wounds exhibit swifter healing with fewer complications compared to open wounds has been a cornerstone of general surgery [6].

Given their superior ability to replace the lost soft tissue structures and instigate tissue formation at the target site, mucosal grafts stand as an almost ideal choice for reconstruction. However, harvesting the required quantity of grafts is substantially difficult, owing to the limited availability of oral mucosa for grafting. Even if available, there is still an elevated likelihood of the risk of scarring at the donor site [6, 18, 19].

Although the removal of viable uniform graft with microtome is a costly option [6], the utilization of skin graft also presents itself as an alternative approach carrying a high likelihood of retaining skin color and also failing to imitate the texture and resiliency akin to that of oral mucosa, which makes the xenogenous collagen graft look at it [8, 20]. As the dermis is primarily composed of collagen, giving an option for the xenogenous collagen graft [7, 21].

Addressing multiple aspects of the healing process, collagen wound dressings offer a superior approach to wound care by effectively replacing the collagen broken down by matrix metalloproteinases, promoting faster wound closure. It also creates optimal conditions for healing, being impermeable to bacteria, maintaining a moist wound environment, and absorbing exudates. Additionally, they help to drift keratinocytes, fibroblasts, and macrophages, interacting with the various regenerative processes, such as angiogenesis and reepithelialization, leading to accelerated and more successful wound closure [22, 23, 24].

Collagen sheets offer several advantages as wound dressing materials for precancerous and cancerous lesions of the oral soft tissues. They are readily available, easy to apply, and well tolerated by oral tissues. Additionally, collagen sheets eliminate the need for a second surgical procedure to harvest a graft or detach a pedicle, reducing morbidity and donor‐site complications, along with possessing favorable attributes including good conformability in mucosal lining, yielding positive clinical assessment regarding its suppleness, resiliency, and ability to mimic the oral wound and surrounding normal tissue, resembling a suitable dressing for the purpose. Furthermore, their use has shown no significant adverse effects.

However, collagen sheets also have some limitations. The application process can be time‐consuming, and although they are relatively affordable, their availability may still vary in certain regions.

The use of collagen sheets is contraindicated in certain patient groups, including ASA Class 3 and 4 patients, individuals on long‐term steroid or antiplatelet therapy, those undergoing radiotherapy, patients with active infections, and pregnant individuals. Proper patient selection and postoperative monitoring are essential to ensure optimal outcomes when using collagen sheets for reconstruction [25, 26].

Comparison of different options for wound coverage are discussed in Table 1 [4, 5, 6, 7, 8, 10, 13, 15, 16, 22].

**TABLE 1:** Comparison of different options for wound coverage.

Feature	Acellular dermal matrix (ADM) graft	Mucosal graft (palatal) graft	Skin graft	Other new options (e.g., allografts and xenografts)	Collagen sheet
Source	Derived from human or animal skin (e.g., cadaveric skin and pig skin)	Autograft from the patient's palate	Autograft from donor skin (typically thigh or forearm)	Allografts (donor tissue), xenografts (animal‐derived), synthetic grafts (biomaterials)	Bovine, porcine, or human‐derived collagen
Tissue type	Acellular dermis	Mucosal tissue	Skin (epidermis + dermis)	Varies based on type (can be allograft, xenograft, or synthetic)	Acellular extracellular matrix (ECM)
Thickness	Thin (generally around 1–2 mm)	Thin (typically 2–3 mm)	Thick (epidermis + dermis)	Varies, typically thinner than skin grafts	Varies (typically 0.5–2 mm)
Immunogenicity	Low (no living cells, so lower rejection risk)	Low (autograft, so no rejection risk)	Higher (risk of immune rejection for allograft and xenograft)	Varies; allografts/xenografts can have higher immunogenicity	Low (highly purified and processed)
Pain postoperative	Minimal (no donor site required)	Moderate (donor site pain)	High (donor site pain, especially with larger grafts)	Variable (depends on graft type)	Minimal (no donor site required)
Healing time	Relatively fast, 1–2 weeks	Moderate, 2–4 weeks	Longer, 3–6 weeks (due to both donor site and graft site healing)	Varies (may be faster than skin grafts)	2–4 weeks (faster epithelialization)
Graft stability	High, as ADM integrates well with surrounding tissues	Moderate to high, as it integrates with the mucosal layer	High, but requires proper vascularization for success	Varies (dependent on graft type, often depends on integration and revascularization)	Moderate to high
Aesthetic outcomes	Good, natural appearance (similar to mucosal tissue)	Good, as it comes from the same region (oral mucosa)	Poor to moderate (distinct difference between skin and mucosa)	Varies (more aesthetic than skin grafts, but less predictable than autografts)	Supports soft tissue healing with minimal scar
Usage	Typically for soft tissue augmentation, covering exposed roots, etc.	For gingival coverage, recession correction	For significant soft tissue loss or large defects	Can be used for soft tissue augmentation, large defects, or when autografts are not possible	Soft tissue regeneration, guided tissue regeneration, wound dressing
Cost	Moderate to high (depending on source and processing)	Low (autograft)	Moderate to high (if donor skin is involved)	Varies (can be expensive, especially for synthetic or processed grafts)	Moderate to high
Indications	Periodontal defects, soft tissue augmentation, root coverage	Root coverage, gingival augmentation	Large defects, severe tissue loss	Periodontal defects, mucosal coverage, bone grafting (e.g., in conjunction)	Periodontal regeneration, mucosal defects, soft tissue grafting

## 5 Conclusion

Though not being an ideal graft, the ease of application in the oral cavity at chairside and good biocompatibility make it a potential alternative as a biological dressing material in the oral cavity. It rather serves as a feasible substitute for an autologous graft but not a replacement. Ultimately, it stands as a valuable addition to the armamentarium of maxillofacial surgeons, providing a satisfactory option for specific clinical scenarios.

## Author Contributions

**Niroj Khanal:** conceptualization, investigation, methodology, project administration, writing – original draft, writing – review and editing. **Bibek Kattel:** methodology, writing – original draft, writing – review and editing. **Mehul Rajesh Jaisani:** supervision, visualization, writing – review and editing. **Siddhartha Rai:** investigation, project administration, supervision.

## Consent

Written informed consents were obtained from all three patients to publish this report in accordance with the journal's patient consent policy.

## Conflicts of Interest

The authors declare no conflicts of interest.

## Data Availability

All data underlying the results reported in this case report are included within the manuscript and no additional source data are required.
